# Taking part in a pharmacogenetic clinical trial: assessment of trial participants understanding of information disclosed during the informed consent process

**DOI:** 10.1186/1472-6939-14-34

**Published:** 2013-09-11

**Authors:** Diana Rose, Jasna Russo, Til Wykes

**Affiliations:** 1 Institute of Psychiatry, King's College London, de Crespigny Park, London SE5 8AF, UK

## Abstract

**Background:**

This study is the first to examine the understandings that participants have of the consent process in a pharmacogenetic trial of anti-depressant medication.

**Methods:**

This was a qualitative cross sectional study. There were 76 participants residing in London, Mannheim, Arhuus and Poznan.

**Results:**

Only one quarter of participants (none in Poznan) could articulate the concept of pharmacogenetics. Heritability and testing medication were also given as the purpose of the trial. Most participants had not appreciated harms that could derive from the trial. Even when shown the consent sheet, participants were confused about DNA profiling. There was evidence that participants appreciated weekly contact with researchers. Most said they would participate in a trial again but would like choice over the intervention they were assigned to.

**Conclusion:**

Participants in this study showed a poor level of informed consent. Although this is not the first time this argument has been made, it is in the case of a pharmacogenetic trial. Further work should investigate the associations between extraneous factors such as information and social support on beneficial or untoward outcomes of antidepressant treatment.

## Background

We have come to rely on clinical trial data to provide the evidence base for treatments. But what do participants think about being involved in trials and could their perceptions have an affect on outcomes? In this study, we examine the perceptions of people with a diagnosis of depression who took part in a pharmacogenetic trial of anti-depressant medication.

The views of participants in trials have received some attention in general medicine. Ellis [1] carried out a systematic review of both doctors’ and patients’ attitudes to Randomised Controlled Trials in the field of cancer focusing in particular on randomisation and informed consent. Ellis argues that randomisation is a hurdle to recruitment as potential participants want choice over treatment and that comprehension of information is often low. The McArther group [2,3] argue that participants in trials suffer from a ‘therapeutic misconception’ when they fail to distinguish clinical care from research and believe that participation in research comprises individualised treatment.

It is often argued that psychiatric patients have impaired decision making capacity which poses special hurdles in obtaining informed consent. This is particularly so in neurodegenerative conditions of the brain such as Alzheimer’s disease and there have been recent calls for clarification on the issues of proxy and surrogate consent [4]. However, it has also been argued that other psychiatric diagnoses can lead to impaired decision making, including schizophrenia and depression. Nevertheless, in a series of studies, Roberts interviewed people with the condition most associated with impaired decision making, schizophrenia, and found no difference between their comprehension of the procedure of the trial and that of people without a psychiatric diagnosis (summarised in Dunn and Roberts [5]). We consider impaired capacity in depression in the Discussion section of this paper.

As far as we can ascertain, there have been no empirical studies of the perceptions of participants in pharmacogenetic studies in psychiatry although there have been conceptual reviews [6-8]. Issues of informed consent in mental health studies are unlikely to differ between pharmacogenetic and other studies as there remains a focus on capacity and vulnerable individuals. Informed consent entails understanding the purpose of a study and achieving it in pharmacogenetic studies may be more difficult as the concept is not easy to grasp. Indeed it is not easy for members of the general public or some policy makers to grasp [9].

For pharmacogenetics studies there is also another important consideration for participants and that is tissue banking and DNA profiling. Two conceptual reviews [6,7] discuss issues such as the retention of samples for further research, the problem of whether or not results should be disclosed to participants, the interests of families and discrimination. In the present study, samples of blood were drawn, were anonymised and participants assured of confidentiality. However, they were aware that their blood samples would be used for research and stored centrally in the main study site.

### Objectives

1. To assess trial participants’ understanding of information during the informed consent process of GENDEP.

2. T o investigate the correspondence between the aims of scientists and the understanding of participants in GENDEP.

### Context of the study

The study reported here was part of a wider investigation. GENDEP is a multi-disciplinary programme of research including a human study of the pharmacogenetics of anti-depressant medication. Other parts of the study included in vitro work and work on animal models. The human part was an open-label partially randomised trial. It took place in eight European countries [10] (for general background to GENDEP see: http://gendep.iop.kcl.ac.uk/background.php).

The funders of GENDEP, with strong support from the main study, required a component of the research to be its Ethical, Legal and Social Implications (ELSI). The participants’ perceptions work was carried out within the Service User Research Enterprise (SURE) who undertook focus groups with service users as well as the study reported here. Essentially, the ELSI research ‘piggy backed’ on the main human study.

The GENDEP main human study took place in nine sites (2 in Germany) and the ELSI sites were chosen to be representative of these. They were: London UK; Mannheim Germany; Aarhus Denmark and Poznan Poland.

## Method

### Study design

This was a cross-sectional qualitative study.

### Study sites

The study sites were London UK, Aarhus, Denmark, Mannheim, Germany and Poznan, Poland. There were 9 study sites in the main study and the ELSI study sites were chosen to be representative of these, including the inclusion of a former Soviet Bloc country and new entrant to the EU (Poland).

Health service configuration differed slightly amongst the sites chosen:

London: limited availability of psychological therapy in public health services.

Mannheim: no additional treatment available apart from medication except in private practice.

Aarhus: Availability of CBT for all people diagnosed with depression although long waiting lists.

Poznan: Limited pharmacopeia available outside the trial and no psychological treatments available in the health services. Most health services must be paid for.

In all countries there was some charge for medication although the amount varied. The drugs for the GENDEP trial were provided free of charge by a pharmaceutical company.

### Study population and sampling framework

The sampling framework was all participants who had taken part in GENDEP in the four ELSI sites at the point of receipt of ethical approval. All eligible participants were sent a letter from the main study researchers and the letters gave contact details of the interviewer for those interested in taking part in the ELSI interviews. The ELSI researcher then made an appointment for interview at a venue of the participant’s choosing.

### Study period

Data were collected in London between January and August 2006; in Aarhus between January 2007 and August 2007; in Mannheim between July 2006 and January 2007; and in Poznan in March and April 2007. All transcriptions were complete and checked at the end of 2007. The London data was analysed in London in 2008, the Mannheim and Poznan data in Berlin during 2008 and 2009 and the Danish data was analysed in London in 2009.

### Data collection

Data collection was by semi-structured, individual, face-to-face interviews. The interviews were audiotaped, transcribed verbatim and translated into English. One researcher was bi-lingual and carried out interviews in both English and German in London and Mannheim. The same researcher worked in Poznan with an interpreter. In Aarhus a specific researcher was employed and the translation into English was carried out by students at the local university.

The interviews attempted to elicit a complete picture of how study participants viewed the experience of taking part in GENDEP. For purposes of this paper, we will chiefly focus on issues of informed consent in the context of pharmacogenetic studies. The key question headings are:

For ability of articulate the concept of pharmacogenetics:

What was the purpose of GENDEP?

For eliciting appreciation of what was consented to:

What did you consent to? (the participant was shown a copy of the consent form, which they had signed, at this point)

For understanding blood being drawn for purposes of genetic research:

Why do you think the researchers took the blood samples? (again a copy of the consent form was presented)

For absence of choice in medication prescribed (NB this was an open-label only partially randomised trial so it would have been inappropriate to probe for understanding of randomisation):

How did you feel about not having a choice in the medication prescribed for you?

General retrospective views:

Looking back, how did you feel at the completion of the study?

All the data were collected by researchers who had had experience of taking anti-depressant medication as there is evidence that respondents are more comfortable in such a situation [11].

### Data analysis

The analysis of all data was conducted in London by service user researchers. The analysis was a thematic one [12] aided by the qualitative software NVivo2 [13]. Thus the approach was deductive, developing themes from the conceptual framework and interview topic guide. The software was used to index, store and retrieve data and delineate the main patterns of meaning contained within the transcripts. Since the interview was clearly structured we did not try to identify cross-question themes. Rather, we shall present the results according to the main headings in the interview. This, of course, is still an interpretive and reflexive procedure as themes never simply ‘emerge’ from data [12]. To preserve the anonymity of participants we shall specify only site and not give any demographic information.

The London researchers who were analysing the UK and Danish data held weekly meetings with the senior researcher (DR) for which they produced NVivo reports. These were checked and any necessary adjustments to coding categories and coding assignments made until thematic saturation was reached. The researcher analysing the German and Polish data produced reports for the senior researcher by email, telephone conferences were held and two face-to face meetings were possible, one in Berlin and one in London. Reflexivity was an important part of these meetings hinging particularly on researchers’ own experiences of taking medication whilst obviating those that were idiosyncratic.

The presentation of results includes verbatim quotations and these were chosen to be representative of the transcripts as a whole.

The study encountered some problems with ethical procedures and this sometimes led to long intervals between completion of the human study and the ELSI interviews. This raised a concern that those with a very long delay did not remember the trial very clearly simply because it was a long time before. We therefore compared three participants each in London, Mannheim and Aarhus with short delays and three in each country with long delays with respect to: i) their understanding of the purpose of the trial; ii) their understanding of what they had consented to and iii) their views on DNA testing. We did not include participants from Poland in this analysis as they all had long delays and, as will be seen, their responses differed from those in other sites.

### Ethical considerations

The main GENDEP study received Ethical Approval from the Institute of Psychiatry Research Committee on 2nd June 2004, Approval Number 292/03. In Aarhus approval was received from The Ethics Committee For Aarhus on 1st July, 2004, approval number: 20040111; in Mannheim, from the Medical Ethics Commission at the University of Heidelberg on 2nd June, 2004, approval number: 24/04; in Poznan from The Karol Marcinkowski Medical University of Poznan Bioethics Commission, 6th October, 2006, approval number: 1137/06.

Potential participants were sent an information sheet prior to the interview. At the start of the interview, the interviewer made sure that the participant understood the information sheet and then signed consent was taken. In terms of potential harms, it was possible that participants might become distressed during the interview and they were told they could stop at any point or take a break. It was also made clear to potential participants that the interview study was independent of the main pharmacogenetic trial and that no data would be transferred between the two studies. This was to ensure that participants felt confident in expressing their views, knowing that the information would not be disclosed to anyone involved in their present or future care.

## Results

### Sample

Seventy six participants agreed to take part in the post-study interviews. Table 1 shows the participation rates.

**Table 1:** Response rates

	**Contacted**	**Interviewed**	**Response rate**
**London**			
Completers	25	16	64%
Drop outs	7	2	29%
**Mannheim**			
Completers	42	16	38%
Drop outs	31	3	9.7%
**Aarhus**			
Completers	40	19	47.5%
Drop outs	0	0	
**Poznan**			
Completers	100	15	15%
Drop outs		5	5%

The median time between completion of the study and interviews with the ELSI team was 8 months. However, in Poznan the delay in gaining ethical approval for the ELSI component meant that most participants had completed the study more than one year before the interview.

A few people in our sample dropped out of the trial part way through and these are also shown in Table 1. We have included them in the analysis as they answered the same questions as the completers and their answers were no different from people who completed the whole trial protocol. There were some specific questions about the reasons and circumstances of discontinuing the study but we will not include them here. The main reason for dropping out was the side-effects of the medication and not process issues to do with the research.

Table 2 shows the mean age and gender distribution across the four sites as compared to the site samples as a whole. There were no significant differences between the main samples and the sub-samples on either of these variables. Of course, demographic representativeness does not mean generalisable perceptions but it does rule out demographic confounders.

**Table 2:** Distribution by age and gender between ELSI sample and total sample at the 4 sites

	**London**		**Mannheim**		**Aarhus**		**Poznan**	
**Age**								
***ELSI sample***								
Mean (SD)	45.92 (11.95)		42.47 (12.35)		39.95 (12.11)		41.89 (12.52)	
N	17		17		19		19	
***Total sample***								
Mean (SD)	44.89 (12.16)		39.91 (11.25)		37.5 (10.96)		39.56 (12.28)	
N	109		106		92		126	
**Gender**								
	M	F	M	F	M	F	M	F
***ELSI sample***	8	10	8	11	4	15	6	14
***Total sample***	39	70	37	33	23	50	19	68
Total	47	80	45	44	27	65	25	82

#### Purpose of GENDEP

We considered this a very important question as without an understanding of the purpose of the study, there can be no informed consent. Some illustrative quotations follow:

“The purpose is to discover – that’s how I understand the purpose I have to say – was to discover how you respond to the medicine you take according to your combination of genes.” (Aarhus)

“The way I got it, it was to clarify, if depressions are genetically hereditary.” (Aarhus)

“As far as I can understand it, what they were trying to find out was what the effect of that particular drug was and what the side effects were and how people reacted to it.” (London)

“From what I can remember it was about testing new drugs. The drugs were allowed to the Polish market by the Ministry but they required some additional testing on volunteers. The doctor said that the study was legal and got full support of the Ministry. That is all I can remember.” (Poznan)

“The first aim of the study was to check my genetic history and whether it could have effect on the history of depression in my family.” (Poznan)

“In my own words, in a nutshell, how does a drug work depending on one’s genetic disposition. So I’d say I basically understood it.” (Mannheim)

No participant in Poznan could explain pharmacogenetics and in fact only a minority (19 people) could do this. The same number believed the study to be about testing medication. A smaller number understood that the study had something to do with genetics but thought it was about heritability.

#### Consent

At this point in the interview, the researcher showed the participant a copy of what they had consented to (the consent form). Many interviewees gave more than one response to this question and, as the quotes in some illustrative quotations show, often responses about risks and consent overlap in the dialogue of the interview. The dialogue illustrates ambivalence and this is not specific to this population but occurs in everyday discourse [14]. However, it was clear that about half of respondents were aware of risks when they signed the consent form. This, of course, means that half were not. Some illustrative quotations follow:

“Well, I knew that I consented to being included in the study. But I cannot tell I knew what it was all about and still don’t know.” (Poznan)

“I’ve read everything very thoroughly before I signed anything and said “I won’t be injected with anything, I won’t take any other drugs, only those prescribed by Dr X [*the treating neurologist*].” And he [*researcher*] said “Ok, ok.” (Mannheim)

“But you know I could get run over crossing the road, you know there’s a bigger risk crossing the road than there is something going wrong in a drugs trial. So life is about risks, that’s not something that really bothers me.” (London)

I: *Were you told about any risks involved with taking part in the study?*

P: “No. I know that side effects can occur after any drug but nobody told me anything special about the drugs.” (Poznan)

I: Did anybody inform you about the risks?

P: “Yes. And that there probably would be hardly any. That the drug has been used for years in hospitals. That it would be pretty much the same basically if I got one or the other.” (Mannheim)

#### DNA/blood sampling

In this part of the interview, the interviewer once again showed the consent form to the interviewee as an aide-memoire. Despite the increasing concern of bioethicists, participants in our study mostly did not object to having blood taken. This is the one question where a substantial number of participants could not remember the reason for the blood being taken. Others were not concerned about it, even if they did understand that it was to be used to collect information about their DNA. Six respondents, across all sites, voiced an initial concern about confidentiality but these fears tended to be allayed when the research team explained that confidentiality was assured. Some illustrative quotations follow:

“I don't think I know what it means. “I agree that a sample of blood can be used to make a cell line in order to provide sufficient DNA for the genetic analysis.” I must have agreed to it.” (London)

I: *Do you recall what you consent to?*

P: “That I participate in the study. And then there’s this study and they collected some blood. That was collected every three weeks, I believe.”

I: You do know, however, that you provided information about your genetics by means of the blood collection?

P: “Yes, precisely, this genetical thingy, yes, it was sent abroad.” (Poznan)

I: Yes, well that’s good. In the next point it says: “I allow, that DNA can be drawn, meaning genomic material from my blood samples, and that this can be used for genetic analysis in the GENDEP-project?”

P: “Yes, but I signed for that somewhere too.”

I: *You did?*

P: “Yes.”

I: *Do you remember?*

P: “No.”

I: *You don’t remember?*

P: “No.” (Aarhus)

“But I remember that it was nothing I had a problem with. It was, just let me think for a moment. I think it was just something like if it was okay that other people looked through my results, people who weren’t the ones doing the interviews.” (Aarhus)

“I didn’t question it very critically then. But I did ask my GP and he said those data were treated very confidentially and wouldn’t be published, well maybe that’s the wrong word, but that there wasn’t any danger that the employer or health insurance got access to the data.” (Mannheim)

#### Lack of choice of medication

This was an open-label part-randomised trial so we cannot determine if our participants understood the principles of randomisation. However, the literature shows that it is choice and decision making that is focused on by participants in trials, at least in trials of cancer treatments [15]. We therefore asked our participants what they felt about not being able to choose the anti-depressant they were assigned. The main category of response was that doctors always choose medication and so it is not surprising that they did in this study too. This was underpinned by two conceptualisations. Participants trusted their doctors and this reference to trust should not be underestimated as it occurred frequently throughout the transcripts. Others were more cynical, saying there is nothing a patient can do – that doctors always decide. Some illustrative quotations are given. It can be seen from the quotation from Poznan, that some physicians were giving participants information about the two medications and saying that they chose which one to prescribe according to the patient’s perceived problems. However, lack of choice was less of a problem in this study than indicated by Ellis [1]. Nonetheless, and somewhat tangentially, we did ask our participants about their preferred treatment for depression and only 11 out of 76 said that this was medication alone, although a combination of medication and talking treatments was the most commonly given option. Some illustrative quotations follow:

“Yes, you might say, that I trusted the professionalism of it. I assumed, my subconscious mind did, actually in the beginning I just let myself drag along, but it had something to do with, that it was professionals in here.” (Aarhus)

“I knew that because I asked if I might choose. I simply got “no” for an answer but then again I’m happy with what I’m given.”

How did you feel regarding the fact that you weren’t allowed to choose?

“Actually bad, but I though to myself the doctor won’t tell me to take anything that’s bad for me, I can rely on that, but I would have liked to have a choice, though.” (Mannheim)

“After some consideration the doctor suggested which of the drugs will be better for me. He introduced me briefly to both of them and said that the one he selected for me would be more suitable for me.” (Poznan)

“That was something that was settled with a draw, as far as I remember, meaning that it was random, so it wasn’t really a demand I made, because I wanted to join, it was just a crucial factor to me, deciding if I wanted to join or not, because you can’t really meet a demand, when it’s something that happens randomly, and then it was kind of lucky.” (Aarhus)

#### Completion of GENDEP

The majority of participants in all sites said they enjoyed participating in GENDEP. Words such as ‘sad’ or ‘disappointed’ were commonly used about the trial coming to an end. However, the reasons for enjoying the trial differed between the sites. Over two thirds of our participants in London, Mannheim and Aarhus mentioned the trial researcher as the reason they felt positive about the study. They appreciated the weekly contact and the pleasant demeanour of the trial researcher. Even with sensitive subjects – such as sexual side-effects of medication - they felt comfortable.

References to the relationship with the researcher were most marked in Mannheim. Only two people did not mention this. Participants talked about this at great length:

“One felt cared for, I could talk to her [*the researcher*] although there were two or three others sitting there. I didn’t have a problem talking about my problems, whether it’s something sexual, I could just talk about it. I just felt protected and great. And the improvement that I had to - change your attitudes and everything where one says “take it easy”, I’m sure the study aimed at that. In how far the drug influenced that I can’t tell. Some doctors also told me I’d be taking some dextrose, well ok. Might be, but I felt better.” (Mannheim)

This quotation is interesting as it implies that weekly contact with the researchers was as important as medication in the relief of depression. However, this should not be overstated. Especially in London, participants were often vague about why they felt disappointed that the trial had come to an end.

In Poznan, the reason for being disappointed when the study ended was quite different. There were no references to good relations with researchers, indeed some participants said they felt like ‘guinea pigs’ and that the doctors were not interested in them personally. The problem for participants in Poznan who had been assigned to the new generation drug was that they had to discontinue it at the end of the study. This was on grounds of expense. The old generation medication was free in that country but participants felt forced into taking it or discontinued medication altogether.

A large majority of participants said they would take part in another clinical study. This was the part of the interviews where issues of ‘altruism’ were apparent. As to recommending to someone else participating in a trial, the majority also said they would do this although many thought that the person should make up their own minds in light of full information.

#### Memory

There were no discernible differences in understanding of the purposes of the trial with respect to the length of time between completion and ELSI interview. Further, the sub-sample of transcripts mirrored the whole data set in this. Similarly, there were no differences in appreciation of harms depending on whether the delay had been short or long between study completion and interview and again the sub-sample reflected the total data set. The sub-sample also mirrored the main sample with respect to DNA profiling. Most did not recognise the clause on the consent form but neither were they concerned when prompted and there was no difference between those with a short and those with a long delay between completion of the trial and ELSI interview.

## Discussion

Apart from the rather different work of Roberts [5], this is one of the first studies to elicit the first hand accounts of understandings of participants in a trial of a biomedical intervention in psychiatry. Certainly it is the first to investigate this in pharmacogenetics. Although pharmacogenetics is a difficult concept to grasp, it was not beyond the understanding of some of our participants, albeit a minority. Proper informed consent means that care should always be taken to ensure that participants understand what a trial involves so as to preserve their autonomy in coming to a decision about whether or not they wish to take part.

This raises again the issue of informed consent and decisional capacity in research studies which we touched on in the introduction. There is a limited literature on decision making capacity and depression. Rudnick [16] suggests that the predominance of cognitive tests in instruments such as the various versions of the MCCAT [17] underplay emotional factors which may impair decision making in depressed patients for *treatment*. Elliot [18] makes a similar argument for the capacity to participate in research. However, both these papers concern severely depressed individuals and the participants in our study were not eligible if they met diagnostic criteria for major depression. The inclusion criteria specified ‘mild to moderate depression’. The McArthur group itself [19] carried out an empirical study with moderately depressed women attending for outpatient psychotherapy and found a high level of capacity when utilising the research version of the MCCAT (MCATT-CR). Although this does not invalidate the arguments of authors such as Rudnick or Elliot, the overall literature suggests that decisional capacity and therefore the capacity to give informed consent is not impaired in the group who participated in GENDEP. Therefore, their recollections and views must be taken at face value.

In terms of our second aim, it is clear that the understandings of those who participated in GENDEP did not correspond to the aims of the scientists who conducted the study. Many thought the study was about testing medication or heritability, that there were no risks and were unclear about tissue sampling. There is evidence also of the ‘therapeutic misconception’ [2,3] and the view that participants should be able to choose the intervention they receive thus indicating that randomisation remains a hurdle to participation in trials [1]. These are not new findings but this study sets them in a new context and one which is likely to become more common as complex biomedical interventions are trialled. Still less is this a critique of GENDEP in particular but is likely to be true of any study involving human participants in any clinical trial. Nevertheless it is incumbent on researchers to make every effort to ensure an adequate understanding. If information sheets or informed consent procedures are not adequate this is a failure of the ethical approval system.

Multi-site studies face particular difficulties. Language is an issue but there is also the problem that different countries and cultures will have different ethical requirements and arrangements as well as different health service configurations. The latter affect attitudes to both conducting and participating in studies such as GENDEP.

Our participants had difficulty understanding the information and consent sheets and this certainly is not specific to this study. Various methods have been proposed to increase comprehension [20]. One method that has not been used, and which we would propose, is to have lay persons involved in drawing up such documents. This has just commenced in mental health in the UK [21]. Researchers who have experience of the diagnosis under consideration could also be deployed, as was done here. Further research could also take the form of a trial of re-presenting the information and consent sheets to randomly allocated participants to see if an additional ‘dose’ improved their understanding. This is consistent with the arguments of Allmark and Mason [22] who used continuous consent to parents in a trial of Total Body Hypothermia (TOBY) for extremely premature babies.

### Limitations

There was a long delay between completing GENDEP and being interviewed about it for some participants and this could affect their original understandings or provoked revisions in the meantime. However, we found no differences between those who responded a short time and a longer time since completing the main study on the main concepts of interest. Moreover, participation in GENDEP was a very intensive procedure. There was little evidence that participants had simply forgotten it. Participants met with researchers every week for 12 weeks for an interview that could last for up to two hours. Follow-up interviews were conducted six months post-study. In addition, blood was taken every 3 weeks. Our sub-study analysis suggests that the various understandings of the trial and its implications for harm and tissue sampling were not affected by the long interval between trial completion and ELSI interview. The ELSI part of the GENDEP study was set up to be independent of the main human study and there was no transfer of individual data between the two investigations. Participants were assured of this. This has the consequence that we have no information about participants’ psychiatric condition that might have compromised their ability to consent although we have argued that this is unlikely in this group. In addition, consent would not have been taken by the main study investigators had capacity been in doubt.

### Future work

A further consequence of the separation of the two investigations is that we have no information about clinical outcomes for our participants and so cannot correlate perceptions with benefit or harm. A major question that arises from this research is whether the factors we have identified make any difference to outcome. Do people who understand what the trial is about and understand what they have consented to achieve a better response to the biomedical intervention? Does the social context make a difference? There is some evidence in our study that intense contact with another person (the researcher) might have had an impact on the relief of depression. Future trials might correlate interview data such as that presented here with outcome data from biomedical interventions in trials to see whether extraneous factors might be causative in amelioration of problems.

GENDEP investigated a biomedical intervention, comparing two anti-depressant medications. Much emphasis today is placed on ‘translational’ research whereby biomarkers are identified in order to develop better treatments. Only 11 out of 76 participants said that their preferred treatment for depression was medication alone, although a combination of medication and talking treatments was the most commonly given option. So in addition to the ethical concerns raised by this study it has also raised a further issue about the acceptability of the outcomes of this research. Biomedical researchers will need to be alert to the priorities of patients as well as researchers when embarking on translational research.

## Conclusion

Participants in this study showed a poor level of informed consent. Although this is not the first time this argument has been made, it is in the case of a pharmacogenetic trial. Further work should investigate the associations between extraneous factors such as information and social support on beneficial or untoward outcomes of antidepressant treatment.

## Competing interests

The authors declare that they have no competing interests.

## Authors’ contributions

All authors were involved in the conception of the study. JR collected and analysed data under the supervision of DR. DR drafted the final manuscript and JR and TW were involved in its critical revision before submission. All authors read and approved the final manuscript.

## Pre-publication history

The pre-publication history for this paper can be accessed here:

http://www.biomedcentral.com/1472-6939/14/34/prepub
